# Nitric oxide positively affects endometrial receptivity via FAAH and NAPE-PLD *in vitro*

**DOI:** 10.1530/RAF-20-0035

**Published:** 2021-04-21

**Authors:** Sarah E Melford, Anthony H Taylor, Justin C Konje

**Affiliations:** 1 Endocannabinoid Research Group, Reproductive Sciences Section, Department of Cancer Studies and Molecular Medicine, University of Leicester, Leicester, UK

**Keywords:** nitric oxide, fatty acid amide hydrolase, N-acylphosphatidylethanolamine, endocannabinoid, endometrial receptivity

## Abstract

**Objective:**

To determine if models of human 'receptive' and 'non-receptive endometrium' differ in their responses to nitric oxide (NO) supplementation by measuring the levels of the enzymes of the endocannabinoid system (ECS) (fatty acid amide hydrolase (FAAH) and N-acylphosphatidylethanolamine-specific phospholipase D (NAPE-PLD)), which control the 'anandamide tone' essential for successful pregnancy.

**Design:**

A study of FAAH and NAPE-PLD expression (using human endometrium) through the menstrual cycle and an *in vitro* using a model of 'receptive' (Ishikawa) and 'non-receptive' (HEC-1A) human endometrial cell lines treated with the NO-donating compound S-nitroso-N-acetylpenicillamine (SNAP).

**Results:**

Immunoreactivity measured by optimised H-score for both FAAH and NAPE-PLD was reduced in secretory (receptive) endometrium compared to proliferative (non-receptive) endometrium (*P* = 0.0009 and <0.0001, respectively). *FAAH* and *NAPE* transcript levels were significantly higher in untreated Ishikawa cells than in HEC-1A cells (*P* = 0.0228 and 0.0001, respectively). Treatment of cultures with SNAP resulted in an increase in the amount of *FAAH* mRNA produced by Ishikawa cells and a decrease in *NAPE-PLD* mRNA. No effect of SNAP was observed in HEC-1A cells. Similarly, FAAH protein was significantly decreased in endometria representative of the receptive endometrium.

**Conclusion:**

These data suggest that NO most likely affects the expression of ECS enzymes in the implantation site of a receptive endometrium; a phenomenon not seen in a non-receptive endometrium. These effects are most marked with FAAH expression, suggesting that FAAH may play the more critical role in ensuring the correct 'anandamide tone' for successful embryo implantation than NAPE-PLD.

**Lay summary:**

Embryo implantation into the wall of the uterus is only successful when the inner wall of the uterus (the endometrium) is ‘receptive’, because if it is ‘non-receptive’, implantation will fail. Previous work showed that enzymes of the 'endocannabinoid system' are critical for implantation by maintaining the correct level of a fat called anandamide. This is by balancing its synthesis (by N-acylphosphatidylethanolamine specific phospholipase D, NAPE-PLD) and degradation (by fatty acid amide hydrolase, FAAH). Using immortalised cell lines as models of ‘receptive’ and ‘non-receptive’ human endometrium, we demonstrate a key stimulator of implantation, nitric oxide, has a positive effect on implantation by both increasing the mRNA levels of the degrading enzyme (FAAH) and decreasing the expression of the synthesising enzyme (NAPE-PLD). These effects are most marked with the degrading enzyme, suggesting that FAAH plays a more critical role than NAPE-PLD in ensuring the correct 'anandamide tone' for successful embryo implantation.

## Introduction

For several decades immense interest has centred on factors that influence the process of early pregnancy events. Numerous factors including progesterone, prostaglandins, homeobox genes, endocannabinoids (such as anandamide), integrins and cytokines have all been identified as playing important roles in blastocyst implantation (Melford *et al.* 2014). Many of these factors interact with each other to either support or hinder successful implantation. For example, progesterone is known to upregulate the production or expression of cyclooxygenase-2 (COX-2), leukaemia inhibitory factor (LIF), integrin β proteins and the homeobox gene A10 (HOX-A10), and causes a fall in levels of the endocannabinoid, anandamide (AEA) at the implantation site (Yu *et al.* 1997, Kozak & Marnett 2002, Karasu *et al.* 2011, Melford *et al.* 2014) – actions which favour successful implantation.

Other endocannabinoids, such as 2-arachidonyolglycerol (2-AG), have also been identified as playing an important role in successful implantation and early pregnancy success, especially in rodents (Habayeb *et al.* 2002, Taylor *et al.* 2007, Sun & Dey 2008, Maccarrone 2009, Bambang *et al.* 2010). This class of molecules (endocannabinoids) are unsaturated fatty acid derivatives of arachidonic acid that act as endogenous ligands for cannabinoid receptors that also bind exocannabinnoids/phytocannabinoids from the *Cannabis sativa* plant. These endocannabinoids, their receptors and the metabolizing enzymes are collectively referred to as the endocannabinoid system (ECS). The main ligands of the ECS are *N*-acylethanolamines (NAEs), of which the most studied is AEA. Other members of the NAE family include *N*-palmitoylethanolamine (PEA), *N*-oleoylethanolamine (OEA), *N*-stearoylethanolamine (SEA) and *N*-linoleoylethanolamide (LEA) (Hanus *et al.* 1993, Wang & Ueda 2009). To date, only AEA has been shown to play a key role in human embryo implantation (Maia *et al.* 2020), however, it is suspected that both OEA and PEA have an 'entourage effect' on levels of AEA (Bambang *et al.* 2010), where these molecules are preferentially degraded by the enzyme fatty acid amide hydrolase (FAAH), resulting in an aberrant increase in AEA concentration at the implantation site causing subsequent miscarriage (Maccarrone *et al.* 2001, Habayeb *et al.* 2008, Taylor *et al.* 2011). The expression of FAAH has been shown to be regulated indirectly by progesterone and Th2 cytokines (Maccarrone *et al.* 2001). *N*-acylphosphatidylethanolamines (NAPEs) are the main precursors of NAEs and are generated by the activity of a type-D phospholipase (PLD), N-acylphoshatidylethanolamine-specific phospholipase D (NAPE-PLD), which hydrolyses NAPEs to NAEs (Guo *et al.* 2005).

It has been shown in murine studies that a careful balance in the activities of NAPE-PLD and FAAH required to ensure that the appropriate levels of AEA are available during implantation (Paria *et al.* 1996, Guo *et al.* 2005). This is referred to as the 'anandamide tone' and is crucial for successful implantation (Wang *et al.* 1999), because it has been demonstrated that higher AEA concentrations inhibited not only the development of mouse two-cell embryos into blastocysts and zona-hatching of blastocysts from eight-cell embryos *in vitro* (Schmid *et al.* 1997), but also that mouse blastocysts exposed in culture to low levels of AEA (7 nM) exhibited accelerated trophoblast differentiation and outgrowth, whereas inhibition of trophoblast differentiation was observed at higher (28 nM) doses (Wang *et al.* 2006). Further support for the notion of 'anandamide tone' being important for embryo implantation comes from the studies of Guo *et al*. (2005) who demonstrated that NAPE-PLD expression was significantly lower at the implantation site when compared to the peri-implantation site, and furthermore proposed that the embryo might play a role in regulating these levels. Although similar studies on human blastocysts have not yet been performed, these data suggest that the 'anandamide tone' might also be important for human implantation.

There is increasing evidence that the interaction between nitric oxide pathways and the ECS play important roles in the success or failure of implantation (Aban *et al.* 2018). While the link between AEA signalling and human implantation is far from fully understood (Maia *et al.* 2020), previous data showed that AEA-induced Ca^2+^ ion signalling in endothelial cells occurs through integrin protein aggregation, a process that is dependent on nitric oxide (NO) synthase activity (Waldeck-Weiermair *et al.* 2008). Similarly, the production of NO is essential to successful human implantation (Khorram 2002) in that increased NO production in the mid-luteal phase acts synergistically with progesterone to enhance receptivity whilst continued expression at the end of the menstrual cycle results in increased endometrial tissue loss through apoptosis and menstruation (Chwalisz & Garfield 2000). These data are similar to the pattern of AEA production in the endometrium, where excess AEA concentrations are associated with implantation failure (El-Talatini * et al.* 2009*b*). Furthermore, NO production and/or release has been shown to influence the activation of CB1 and CB2 (the main receptors involved in the ECS) (Lipina & Hundal 2017) in a variety of tissues, including rat placentae. These data suggest a possible link between AEA signalling, NO signalling, and endometrial receptivity.

We, therefore, set out to determine if the expression of the ECS enzymes FAAH and NAPE-PLD are regulated throughout the menstrual cycle and especially during the ‘window of implantation’, and to examine and compare the effects of NO on the production of *FAAH* and *NAPE-PLD* in a model of human 'receptive endometrium'.

## Materials and methods

### *In vivo* expression of ECS enzymes

Knowing that the expression of NAPE-PLD differs in the implantation and inter-implantation zones of the murine endometrium (Paria *et al.* 1996, Schmid *et al.* 1997, Guo *et al.* 2005, Maccarrone 2009), we first explored the expression of NAPE-PLD and FAAH in the human endometrium. This study was reviewed and approved by the Leicestershire, Northamptonshire and Rutland Research Ethics Committee (LREC #06816). All patients who donated endometrial biopsies gave their informed signed written consent to their tissue being used in this study. Endometrial biopsies were taken throughout the menstrual cycle, and immunohistochemistry performed as we previously described (Habayeb *et al.* 2008, El-Talatini * et al.* 2009*a*) using commercial rabbit polyclonal FAAH antibodies (1 in 2000 dilution; Cat no: FAAH11-A; Alpha Diagnostics Inc., San Antonio, TX, USA) and rabbit polyclonal NAPE-PLD antibodies (1 in 200 dilution; Cat no: ABIN110270, Cayman Chemicals).

**Figure 1:**
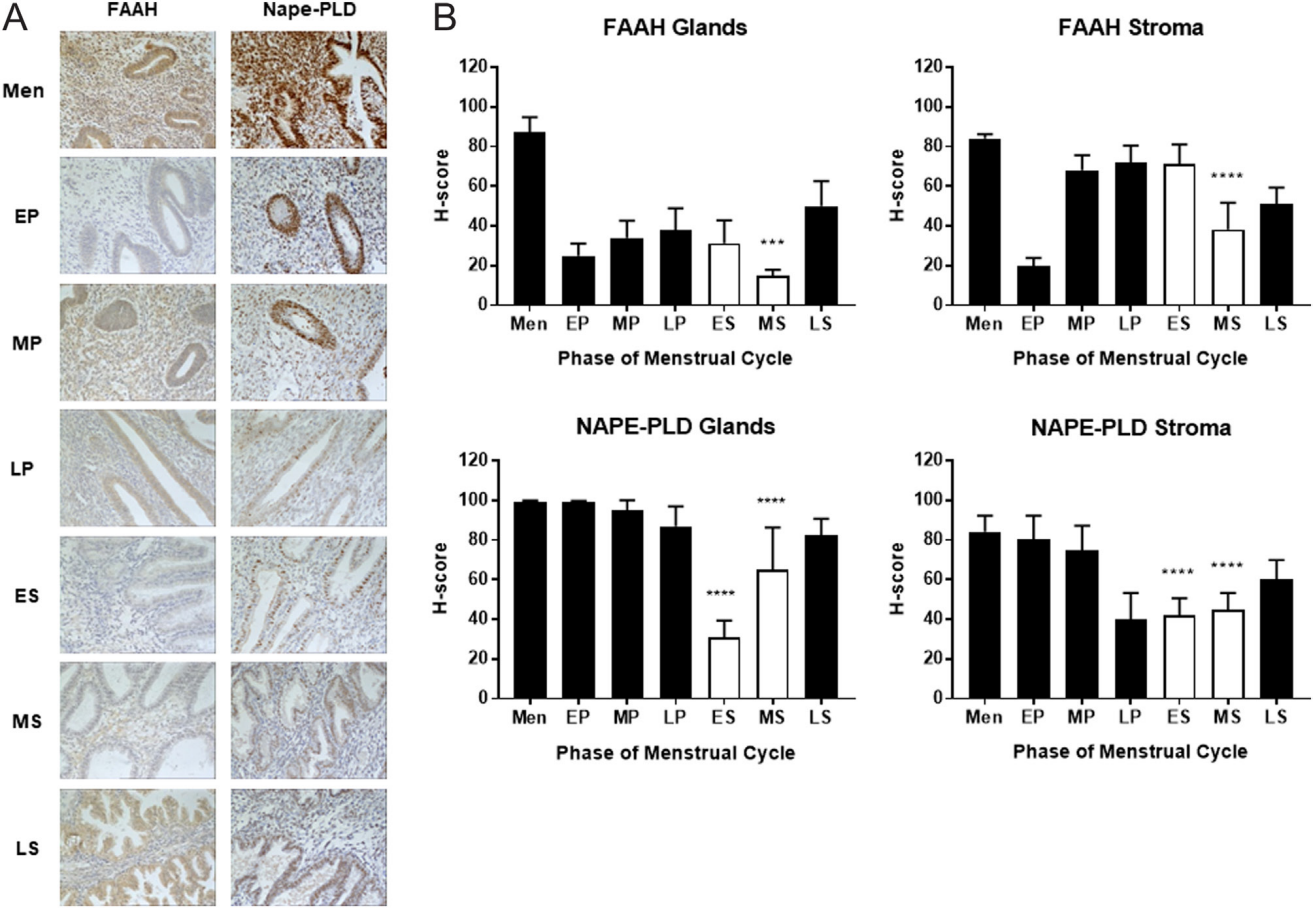
Immunohistochemical staining and histomorphometric analyses of NAPE-PLD and FAAH protein expression through the menstrual cycle. Endometrial biopsies taken from the menstrual (Men, *n*  = 6), early- (EP, *n*  = 8), mid- (MP, *n*  = 8) and late-proliferative (LP, *n*  = 8) phases and the early- (ES, *n*  = 8), mid- (MS, *n*  = 8) and late-secretory (LS, *n*  = 8) phases of the menstrual cycle were subjected to immunohistochemistry with FAAH and NAPE-PLD specific (Panel A) antibodies. After image capture, the intensity of brown DAB staining was determined histomorphometrically (Panel B) for the endometrial glandular epithelial cells (left panels) and endometrial stromal cells (right panels) as separate measurements for each enzyme. The data are presented as the mean ± s.d. histoscore (H-score). FAAH immunoreactivity was reduced in both the glandular epithelial and stromal cells of receptive (open bars) endometria (MS; ***P = 0.0009; ****P < 0.0001; one-way ANOVA with Dunnett’s multiple comparison test) when compared to the H-score of the non-receptive (solid bars) endometria (MP). NAPE-PLD immunoreactivity was also reduced in the glandular epithelial and stromal cell compartments during the receptive phase of the menstrual cycle (ES vs MP ****P < 0.0001; MS vs MP ****P < 0.0001).

Histomorphometric analysis of 10 representative images from each tissue section was performed as we previously described (Gebeh *et al.* 2012) using image analysis software (ImageScope, version 10.2.2.2319; Aperio software distributed by Leica Microsystems). The number of samples used in each phase of the cycle are listed in the legend to Fig. 1.

### Choice of receptive and non-receptive human endometrial cell lines

To simulate a 'receptive' human endometrium, the Ishikawa cell line (an endometrial adenocarcinoma cell line) was chosen (Castelbaum *et al.* 1997, Hannan *et al.* 2010) because it has features possessed by receptive endometrial cells *in vivo* including the expression of oestrogen, progesterone, androgen and luteinising hormone receptors (*in vivo* endometrial cells also express chorionic gonadotrophin) (Hannan *et al.* 2010). In addition, these cells increase their expression of the integrins α2β1, α3β1, α6β4 and decrease that of αvβ3 and the progesterone receptor, in response to oestrogen and progesterone, characteristic of the implantation site of the human endometrium (Castelbaum *et al.* 1997). All these features mean that Ishikawa cells are considered a good model of an adhesive and receptive endometrium (Castelbaum *et al.* 1997).

HEC-1A cells (another endometrial adenocarcinoma cell line) were chosen to simulate a 'non-receptive' human endometrium because they have been identified as being a poorly adhesive cell line (Hannan *et al.* 2010), but otherwise are closely matched to Ishikawa cells in so far as possessing both oestrogen and progesterone receptors (but not androgen receptors) (Horne *et al.* 2006).

### Cell culture

Ishikawa cells (5 × 10^5^) were cultured in 2 mL of DMEM:F12 with glutamax, 10% fetal calf serum and 1% penicillin/streptomycin, while HEC-1A cells (5 × 10^5^) were cultured in 2 mL of McCoy’s 5a Medium Modified with 10% fetal calf serum. Both cell types were cultured independently in an incubator maintained at 37°C with 5% CO_2_ in air, for 24 h to achieve 50% confluence. Once the cells had reached 50% confluence, the culture medium was replaced with the respective medium augmented with different concentrations of the nitric oxide (NO)-donating compound S-nitroso-N-acetylpenicillamine (SNAP; 0–2000 µM, Zhan *et al.* 2018); *n*  = 6 for each concentration used. The cells were then re-incubated for a further 48 h, before their RNA was prepared.

### Total cellular RNA extraction and quantification

Total cellular RNA was extracted using the mirVanaTMmiRNA Isolation Kit (Life Technologies) according to the manufacturer’s protocol and its quantity and quality assessed using a Nanodrop spectrophotometer (Thermo Scientific). Genomic DNA contamination was then removed from each sample using Turbo-DNAse (Thermo Fisher Scientific) according to the manufacturer’s instructions. Next, the RNA concentration was standardised to 10 µg/100 µL and incubated at 37°C for 30 min before the reaction was inactivated (using inactivation buffer supplied in the TURBO-DNasefree kit). RT was performed using a Multiscribe kit (Thermo Fisher Scientific) and the resulting cDNA stored at −20°C.

### Quantitative real-time PCR

The relative levels of cellular transcripts for NAPE-PLD and FAAH were analysed using Taqman probes for human *NAPE-PLD* (Hs00419593_ml), human* FAAH* (Hs01038660_ml) with human *GAPDH* (Hs02786624_gl) as the internal endogenous control (Applied Biosystems, ThermoFisher Scientific) in a StepOne qPCR system (ThermoFisher Scientific). All reactions were in a final volume of 20 µL and consisted of 2 µL of cDNA, 10 mL of Taqman Universal PCR Master mix (Applied Biosystems, and 8 mL of DNase-free water. The reactions were run on a StepOne Plus instrument (Applied Biosystems). The thermocycler conditions were as follows: 2 min at 50°C, 10 min at 95°C, and then 40 cycles of 15 s at 95°C and 1 min at 60°C. All reactions were performed in triplicate (both biological and technical). Cq values from the reactions were taken directly from the PCR machine and Relative Expression calculated using the 2^−ΔΔct^ method (Livak & Schmittgen 2001).

### Statistical analyses

Histomorphometric (H-score) data and enzyme transcript levels are presented as the mean ± s.d. for the indicated number of samples shown in the figure and legends. Statistical differences for the histomorphometric analyses were performed using one-way ANOVA with Dunnett’s multiple comparison test for the expression values through the menstrual cycle and Student’s unpaired *t*-test with Welch correction for unequal variances) when comparing 'receptive' to 'non-receptive' endometria. Statistical analysis for basal transcript levels in the two cell types was performed using Student’s unpaired t-test, whilst significance for the effect of SNAP on FAAH and NAPE-PLD transcript levels was determined using one-way ANOVA with Tukey’s multiple comparison test. Pearson’s linear correlation analysis was used to determine the relationships between FAAH or NAPE-PLD transcript levels and SNAP concentration. In all cases, a *P*-value <0.05 was considered to be statistically significant. All data analyses were performed using Prism version 7:00 for Windows (GraphPad, www.graphpad.com).

## Results

### Expression of ECS enzymes in receptive and non-receptive endometrium – *in vivo* studies

The expression of ECS enzymes were analysed throughout the menstrual cycle by immunohistochemistry, using target-specific commercial antibodies (Fig. 1A). The staining patterns show that changes in enzyme expression occurred during the 'receptive' stage of the cycle (early/mid-luteal phase). FAAH immunoreactivity (measured by unbiased H-score; (Fig. 1B) was reduced in both the glandular epithelial and stromal cells of receptive endometria (mid-secretory phase (MS); *P* = 0.0009; *P* < 0.0001) when compared to that of the non-receptive endometria (mid-proliferative phase (MP)) in glandular epithelial and stromal cells, respectively). NAPE-PLD immunoreactivity was also reduced in glandular epithelial and stromal cell compartments during the receptive phase of the menstrual cycle (early secretory (ES) vs MP *P* < 0.0001; MS vs MP *P* < 0.0001), respectively.

**Figure 2:**
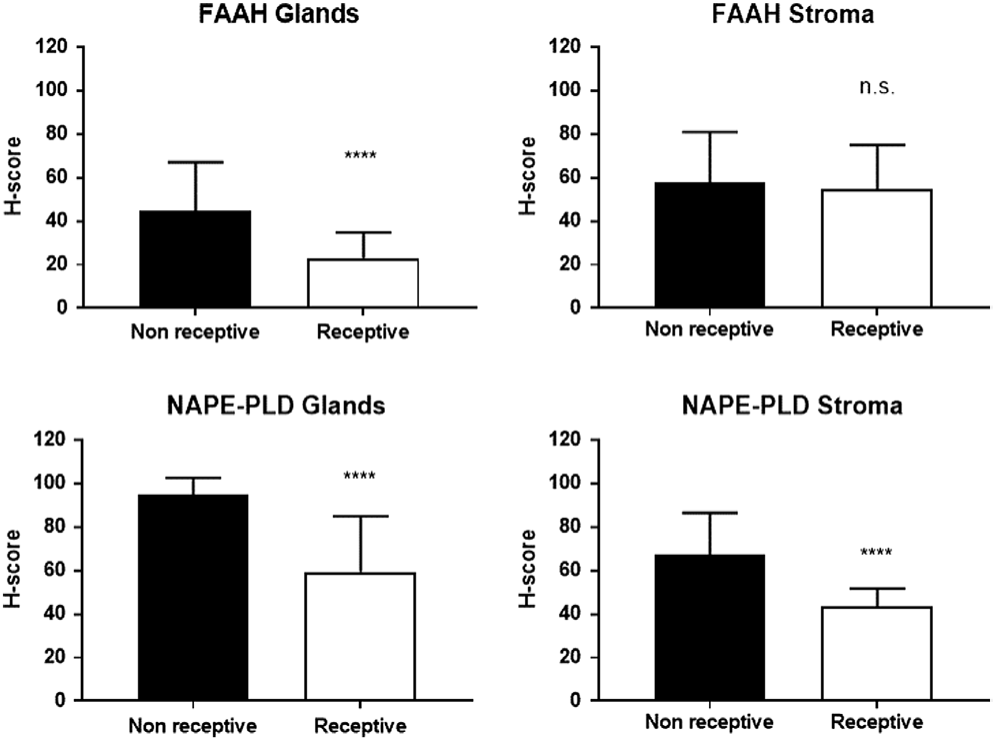
Histomorphometric analyses of NAPE-PLD and FAAH protein expression in receptive and non-receptive endometria. Endometrial biopsies were taken from the early and middle secretory phases of the menstrual cycle (ES and MS = receptive), and biopsies from the proliferative and late secretory phase of the menstrual cycle (EP, MP, LP and LS = non-receptive) were subjected to immunohistochemistry (see Fig. 1) with FAAH (upper panels) and NAPE-PLD-specific (lower panels) antibodies. After image capture, the intensity of brown DAB staining was determined histomorphometrically for the endometrial glandular epithelial cells (left panels) and endometrial stromal cells (right panels). The data are presented as the mean ± s.d. histoscore (H-score). Both FAAH and NAPE-PLD immunoreactivity were reduced in the glandular epithelial cells of receptive endometria (*n* = 16; ****P < 0.0001; Student’s unpaired *t*-test with Welch correction for unequal variances) when compared to that of the non-receptive endometria (*n* = 38). There was no change in the amount of FAAH expressed in the stroma. By contrast, there was a significant reduction in NAPE-PLD expression in the stroma in the receptive endometria when compared to the non-receptive endometria (****P < 0.0001).

When the expression of NAPE-PLD or FAAH was categorised based on whether the endometria were either 'receptive' or 'non-receptive', the same phenomenon was identified (Fig. 2). Both FAAH and NAPE-PLD immunoreactivities were reduced in the glandular epithelial cells of receptive endometria (*P* < 0.0001) when compared to that of the non-receptive endometria. There was no change in the amount of FAAH expressed in the stroma. By contrast, the amount of NAPE-PLD immunoreactivity was significantly (*P* < 0.0001) lower in the stromal compartment of receptive endometria when compared to that found in the non-receptive endometria.

### Transcript levels of the ECS enzymes in untreated Ishikawa and HEC-1A cells

**Figure 3:**
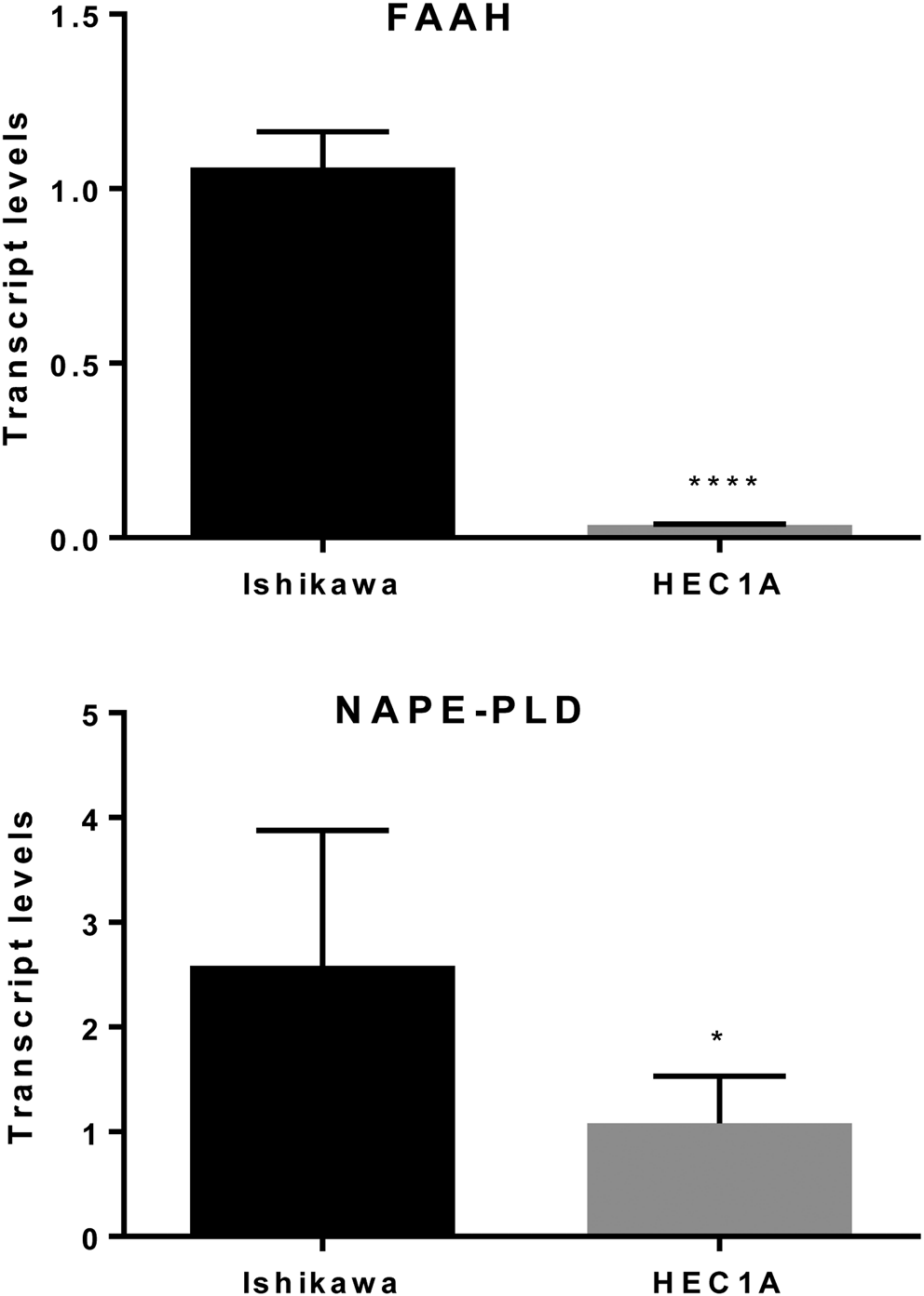
A comparison of the relative levels of *FAAH* (upper panel) and *NAPE-PLD* (lower panel) mRNA in untreated Ishikawa and HEC-1A cells. Ishikawa and HEC-1A cells were cultured for 48 h and total cellular RNA prepared as described in the 'Materials and Method' section. The amounts of *FAAH* and *NAPE-PLD* mRNA generated from RT and by using gene-specific TaqMan based PCR were normalised to mRNA levels of the reference gene *GAPDH* to provide a relative mRNA level. The data are presented as the mean ± s.d. of *FAAH* or *NAPE-PLD* mRNA levels relative to the amounts of GAPDH. Both sets of data showed a significant lower amount of transcripts for the enzymes in the HEC-1A cells (*FAAH* ****P  < 0.0001, *NAPE-PLD* *P = 0.0228; Student’s unpaired t-test) when compared to the levels found in Ishikawa cells.

Having shown that the expression of NAPE-PLD and FAAH differs between the receptive and non-receptive phases of the menstrual cycle, we used the *in vitro* studies to explore the effects of NO on the expression of these two enzymes. First, we measured the relative levels of the transcripts for *FAAH* and *NAPE-PLD* in the two cell lines. *FAAH* and *NAPE-PLD* have been shown to be expressed in endometrial carcinoma (Ayakannu *et al.* 2019) and Ishikawa cells (Fonseca *et al.* 2018). However, it was necessary to confirm that HEC-1A cells also express *FAAH* and *NAPE-PLD* (Fig. 3). The data confirmed that *NAPE-PLD* and *FAAH* transcripts are produced by both Ishikawa and HEC-1A cells, however, the amount of transcripts for both enzymes was significantly higher in Ishikawa (receptive) cells than in HEC-1A (non-receptive) cells, (*NAPE-PLD*, *P* = 0.0228; *FAAH*, *P* < 0.0001).

### The effect of NO supplementation on FAAH and NAPE-PLD transcript levels

**Figure 4:**
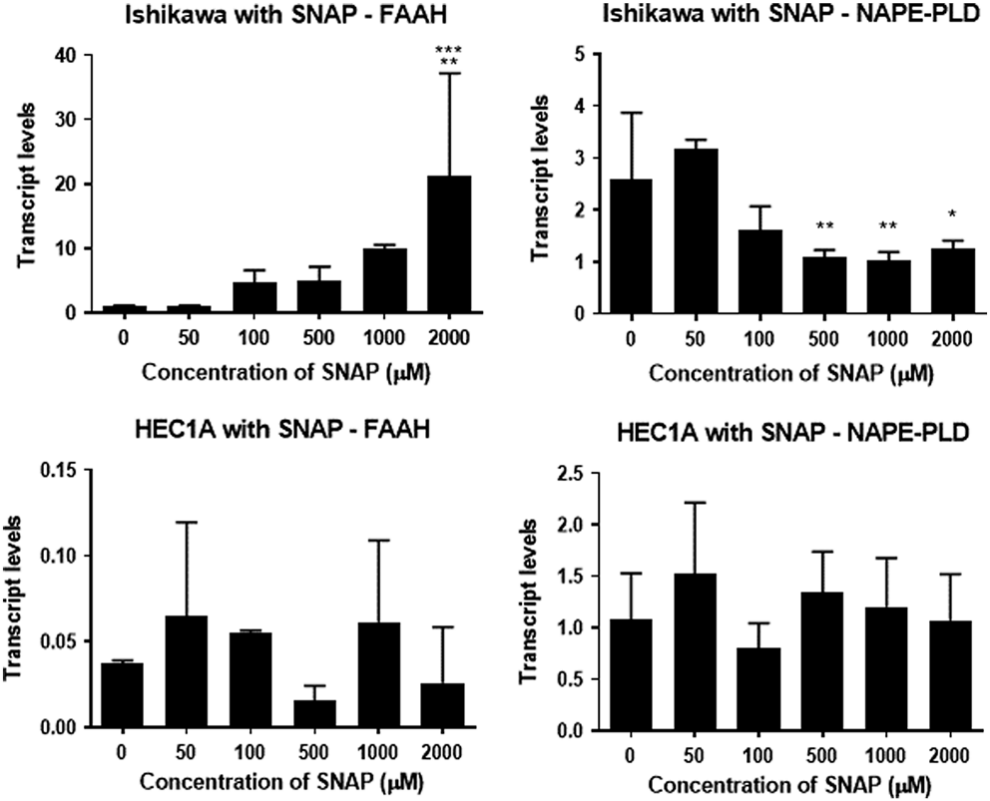
The effect of SNAP on *FAAH* and *NAPE-PLD* mRNA expression in receptive and non-receptive endometrial cell lines. Ishikawa cells (upper panel) and HEC-1A cells (lower panel) were exposed to the indicated concentrations of SNAP for 48 h. The amount of *FAAH* and *NAPE-PLD* mRNA present was determined after RT and by using gene-specific TaqMan based PCR (see Materials & Methods section). For each sample, *FAAH* and *NAPE-PLD* amplicon levels were then normalised against those for the reference gene *GAPDH*. The data are presented as the mean ± s.d. relative amounts of *FAAH* (*n* = 6) or *NAPE-PLD* (*n* = 6) with the error bars not shown when encompassed by the bar. One-way ANOVA (*P* < 0.0001) revealed that *FAAH* transcript levels in Ishikawa cells treated with 2000 µM SNAP was significantly higher than 0, 50, 100 and 500 µM SNAP (***P < 0.001 for 0 and 50 µM, **P < 0.01 for 100 and 500 µM SNAP; Tukey’s multiple comparison test). By contrast, SNAP had no significant effect on *FAAH* transcript levels in HEC-1A cells (*P* > 0.05, Tukey’s multiple comparison test). Similarly, in Ishikawa cells, 500, 1000 and 2000 µM SNAP caused a significant decrease in *NAPE-PLD* transcript levels (**P < 0.01 for 500 and 1000 µM, *P < 0.05 for 2000 µM; Tukey’s multiple comparison test) when compared to the untreated control and 50 μM SNAP. Similar to the data for *FAAH* expression, SNAP had no effect on *NAPE-PLD* transcript levels in HEC-1A cells (*P* > 0.05).

The relative amount of* FAAH* mRNA produced by Ishikawa cells increased in a SNAP dose-dependent manner (Fig. 4), with a significant difference from the untreated control being observed at 2000 µM (*P* < 0.001). Additionally, the highest SNAP concentration (2000 µM) produced a significantly higher level of *FAAH* transcripts when compared to all of the other tested SNAP concentrations (except that of 1000 µM; 0 µM – *P* < 0.001; 50 µM – *P* < 0.001; 100 µM – *P* <0.01; 500 µM – *P* <0.01) suggesting that a maximum effect of SNAP on *FAAH* mRNA levels in Ishikawa cells is somewhere between 1000 and 2000 µM.

This finding was not replicated when treating HEC-1A cells with SNAP (Fig. 4) where there was no effect on the relative amounts of* FAAH* mRNA produced (*P* > 0.05) suggesting that HEC-1A cells produce very little *FAAH* mRNA and the amount of RNA remained constant during treatment with increasing SNAP concentrations.

Treating Ishikawa cells with SNAP also affected the amount of *NAPE-PLD* mRNA with a gradual decrease in *NAPE-PLD* mRNA produced by Ishikawa cells that was significant at concentrations above 500 µM (Fig. 4). There was a significant (*P* < 0.01) difference in *NAPE-PLD* mRNA levels between the non-treated cultures at the lowest concentration of SNAP (0 and 50 µM) and the higher concentrations (1000 and 2000 µM), where SNAP treatment significantly reduced the amounts of *NAPE-PLD* mRNA in relation to both the untreated control and the lowest SNAP dose.

Furthermore, no effect of increasing concentrations of SNAP on the relative amounts of *NAPE-PLD* mRNA produced by HEC-1A cells was observed (*P* = 0.2170) (Fig. 4). The mean relative amount of *NAPE-PLD* mRNA remained close to 1.0 (0.80–1.52), and there was no significant change from this value suggesting that the expression levels of *NAPE-PLD* and *GAPDH* were similar (*P* = 0.1862).

### Comparison of the ECS enzymes ratio in Ishikawa and HEC-1A cells

**Figure 5:**
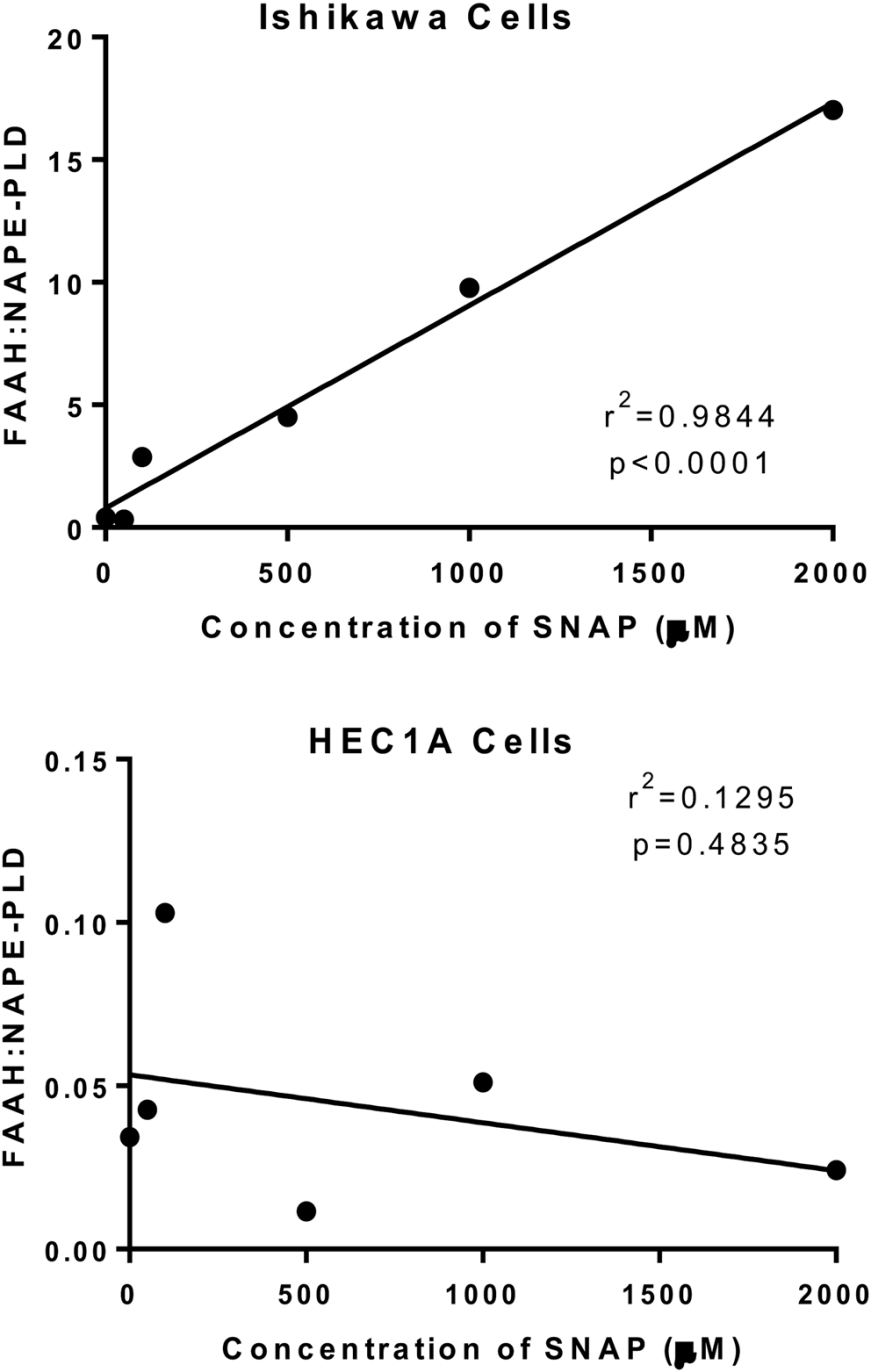
The effect of the NO inducer SNAP on the ratio of *FAAH* to *NAPE-PLD* transcript levels in Ishikawa and HEC-1A cells. The data are presented as a single plot for each *FAAH:NAPE-PLD* ratio for Ishikawa (upper panel) and HEC-1A cells (lower panel) treated for 48 h with the indicated concentrations of SNAP. Linear regression analysis (Pearson’s correlation) revealed a strong correlation between SNAP concentrations and the *FAAH:NAPE-PLD* ratio in Ishikawa cells (r^2^ = 0.9844, *P*  < 0.0001), whereas there was no correlation between the *FAAH:NAPE-PLD* ratio and SNAP concentrations in HEC-1A cells (r^2^ = 0.1295, *P* = 0.4835).

Having identified a strong correlation between the upregulation in the amount of *FAAH* mRNA and downregulation of *NAPE-PLD* mRNA in response to SNAP concentrations in Ishikawa cells, the next step was to examine the possible regulatory mechanism between SNAP treatment and enzyme transcript levels by comparing the ratio of mean relative amounts of *NAPE-PLD* to *FAAH* against SNAP concentration (Fig. 5). The data showed a strong dose-dependent correlation (r = 0.9844, *P* < 0.0001; Pearson’s correlation) for Ishikawa cells, but no correlation for HEC-1A cells (r = 0.1295, *P* = 0.4835) suggesting a common regulatory pathway for both enzymes in the Ishikawa cells but not in the HEC-1A cells.

## Discussion

Several important findings have been described as a result of this work. First, this is the first time it has been shown that HEC-1A cells express both of the ECS regulatory enzymes FAAH and NAPE-PLD. Secondly, while the relative amounts of mRNA were higher in 'receptive' than 'non-receptive' cells, a comparison of the *NAPE-PLD:FAAH* ratio in 'receptive' and 'non-receptive' cell lines, indicated that the amount of *NAPE-PLD* mRNA produced was 10× higher in the non-receptive cells (29.13 in HEC-1A cells) than in the receptive cells (2.43 in Ishikawa cells). Although the activities of the enzymes have not been evaluated, this first may suggest that regulating the 'anandamide tone' is important in embryo implantation because this ratio of the enzymes (and thus the overall concentration of AEA) would result in a higher concentration of AEA in the non-receptive endometrium. Thirdly, is the finding that treatment of Ishikawa cells with SNAP (and its resultant supplementation with NO) affected the amount of both *FAAH* and *NAPE* mRNA produced in a dose-dependent manner. This phenomenon was not seen when the experiment was repeated with HEC-1A cells. When considering endometrial receptivity, these results suggest that there may be an interaction between NO and the enzymes of the ECS in a 'receptive endometria' cell line that is not seen in a 'non-receptive endometria' cell line.

One limitation of the present study was that this work was limited to measuring amounts of NAPE-PLD and FAAH transcripts in the cell lines. A natural progression would be to identify whether NO had the same effect on enzyme protein expression, and if these findings translated into altered enzyme activity. However, such studies are difficult because effective methods for accurate measurement of these parameters do not yet exist (Weller 2016). However, *in vivo* examination of FAAH and NAPE-PLD protein expression using previously validated histomorphometric analysis of FAAH and NAPE-PLD immunoreactivity (Karasu *et al.* 2011, Gebeh *et al.* 2012), indicated that during the window of implantation (ES and MS phases of the menstrual cycle), the expression of NAPE-PLD was decreased in line with the notion that loss of NAPE-PLD expression would result in the observed reduction in anandamide production by the endometrium during the receptive phase of implantation (Paria *et al.* 1996, Guo *et al.* 2005). Although FAAH expression also decreased during these phases, those changes were the reciprocal of the NAPE-PLD expression in the ES and MS phases of the menstrual cycle (see white bars in the gland data of Fig. 1B), suggesting that ECS enzyme activity during the implantation process is complex, but results in a lower AEA concentration supportive of implantation, but not involving the blastocyst as suggested (Guo *et al.* 2005).

From animal studies, it is known that the expression of both NAPE-PLD and FAAH varies between the implantation zone and inter-implantation zone, and this is vital in providing the required 'anandamide tone' to enable successful implantation (Bambang *et al.* 2010). It is also already known that the production of NO regulates various physiological reproductive processes such as implantation, decidualisation and myometrial relaxation (Duran-Reyes *et al.* 1999, Chwalisz & Garfield 2000, Vercelli *et al.* 2009). When considering the link between 'anandamide tone' and NO, it has been shown that high levels of AEA triggered increased NO synthesis (Vercelli *et al.* 2009). The results of the present study suggest that NO may be playing a key role in maintaining this 'anandamide tone' – with low levels of NO encouraging NAE synthesis (by increasing *NAPE-PLD* transcript levels) and high levels of NO encouraging NAE degradation (by increasing *FAAH* transcript levels). It is also noteworthy that the supplementation of NO by SNAP exerted a much greater effect on the relative amount of *FAAH* mRNA by Ishikawa cells than that for *NAPE-PLD* mRNA. While there is no doubt that both enzymes play a key role in maintaining an appropriate 'anandamide tone', these results suggest that FAAH may be more influential in the control necessary for human endometrial receptivity. We believe this is the first documented evidence that NO supplementation may play a role in helping to maintain a suitable anandamide tone essential for human embryo implantation.

An acceptable weakness of our study is that we used monolayer cultures of immortalised endometrial epithelial cells. It would be ideal to use cultured human endometrial secretory and proliferative phase cells as well as primary cultures of cells isolated from both implantation and non-implantation sites taken from human pregnancies, but such cultures are yet to be possible (as far as we are aware). Despite this limitation, we feel confident that our data provide an important observation, which should increase understanding of the modulation of the role of 'anandamide tone' in the regulation of implantation.

## Conclusion

There appears to be a link between the endocannabinoid system and NO when considering human endometrial receptivity, with our results suggesting that an appropriate level of NO may have a role in maintaining an appropriate 'anandamide tone', which is necessary for successful embryo implantation. We have also provided evidence that FAAH appears to be more influential in this process than NAPE-PLD.

## Declaration of interest

The authors declare that there is no conflict of interest that could be perceived as prejudicing the impartiality of the research reported.

## Funding

This work was supported by the National Institutes for Health Research http://dx.doi.org/10.13039/100005622 (NIHR) under their Research for Patient Benefit Programme http://dx.doi.org/10.13039/501100009128 (RfPB) scheme (grant number PB-PG-0909-19305).

## Author contribution statement

All authors had conceptualisation roles. S E M performed the *in vitro* studies and AHT performed the IHC work. All authors analysed the data. S E M wrote the first draft and all authors edited and revised the paper. JCK acts as the guarantor for the study.
